# Bifunctional TaqII restriction endonuclease: redefining the prototype DNA recognition site and establishing the Fidelity Index for partial cleaving

**DOI:** 10.1186/1471-2091-12-62

**Published:** 2011-12-05

**Authors:** Agnieszka Żylicz-Stachula, Olga Żołnierkiewicz, Katarzyna Śliwińska, Joanna Jeżewska-Frąckowiak, Piotr M Skowron

**Affiliations:** 1 Institute for Environmental and Human Health Protection, Department of Chemistry University of Gdańsk, Sobieskiego 18, 80-952 Gdańsk, Poland

## Abstract

**Background:**

The TaqII enzyme is a member of the *Thermus *sp. enzyme family that we propounded previously within Type IIS restriction endonucleases, containing related thermophilic bifunctional endonucleases-methyltransferases from various *Thermus *sp.: TaqII, Tth111II, TthHB27I, TspGWI, TspDTI and TsoI. These enzymes show significant nucleotide and amino acid sequence similarities, a rare phenomenon among restriction endonucleases, along with similarities in biochemical properties, molecular size, DNA recognition sequences and cleavage sites. They also feature some characteristics of Types I and III.

**Results:**

Barker et al. reported the Type IIS/IIC restriction endonuclease TaqII as recognizing two distinct cognate site variants (5'-GACCGA-3' and 5'-CACCCA-3') while cleaving 11/9 nucleotides downstream. We used four independent methods, namely, shotgun cloning and sequencing, restriction pattern analysis, digestion of particular custom substrates and GeneScan analysis, to demonstrate that the recombinant enzyme recognizes only 5'-GACCGA-3' sites and cleaves 11/9 nucleotides downstream. We did not observe any 5'-CACCCA-3' cleavage under a variety of conditions and site arrangements tested. We also characterized the enzyme biochemically and established new digestion conditions optimal for practical enzyme applications. Finally, we developed and propose a new version of the Fidelity Index - the Fidelity Index for Partial Cleavage (FI-PC).

**Conclusions:**

The DNA recognition sequence of the bifunctional prototype TaqII endonuclease-methyltransferase from *Thermus aquaticus *has been redefined as recognizing only 5'-GACCGA-3' cognate sites. The reaction conditions (pH and salt concentrations) were designed either to minimize (pH = 8.0 and 10 mM ammonium sulphate) or to enhance star activity (pH = 6.0 and no salt). Redefinition of the recognition site and reaction conditions makes this prototype endonuclease a useful tool for DNA manipulation; as yet, this enzyme has no practical applications. The extension of the Fidelity Index will be helpful for DNA manipulation with enzymes only partially cleaving DNA.

## Background

Restriction endonucleases (REases) are indispensable as molecular scissors in the analysis, rearrangement, cloning and sequencing of DNA [1-3]. Restriction-modification (RM) systems have been classified into four major types on the basis of their genetic and polypeptide organization, cofactor requirements, and their modes of recognition and cleavage: I, II, III, and IV [4]. Of the 3945 biochemically or genetically characterized restriction enzymes [5] currently known, 3834 Type II RM systems make up the largest biochemically characterized fraction [5]. Only 299 of this vast number, however, are enzymes with different and unique specificities; the remainder are isoschizomers, that is, enzymes with the same substrate specificity as the prototype but originating from different bacterial genera, species or strains [5].

Worth mentioning among these enzymes are the subtype IIS REases, which, in contrast to orthodox Type II REases, interact with the asymmetric sequence and typically cut DNA in a strictly fixed location beyond its recognition site to produce blunt or sticky ends with 3'- or 5'-overhangs [6]. The known prototype subtype IIS specificities discovered to date comprise only a small fraction of the possible asymmetric recognition sequences with no associated specific REase. For this reason, it is highly likely that new prototypical REases will be discovered within this very subtype. Statistically, there are many potential symmetric and asymmetric recognition sites: 256 possible combinations of 4-bp sites, 1024 of 5-bp sites, 4096 of 6-bp sites, 16 384 of 7-bp sites and 65 536 of 8-bp sites. Even with the current advent of whole genome sequencing aided by sophisticated software analysis of the potential restriction endonuclease genes present, accumulation of novel prototype specificities is slow. As REases still form the core of recombinant technologies, new findings of these enzymes in nature or the creation of artificial specificities *in vitro *[7-10] or the improvement of existing ones (such as this work) is of scientific and practical significance. The palette of everyday cloning tools is enlarged with every new prototype that becomes available. Particularly refined improvements and applications of sub-Type IIS enzymes have been pioneered by Wacław Szybalski since the 1980s: these include universal restriction endonucleases, DNA cleavage at a pre-programmed site, gene amplification, gene fusion, unidirectional DNA trimming, methylated base location in DNA and gene mutagenesis using excision linkers [7-11]. The majority of known Type II REases have been isolated from mesophilic bacteria. Such enzymes are stable at temperatures below 45°C and are usually rapidly denatured at higher temperatures. The relatively few restriction endonucleases capable of withstanding higher temperatures therefore represent an extremely useful additional molecular tool [12-14]. For this reason the investigation of existing restriction endonucleases and the search for novel, thermally stable ones is entirely justified.

83 thermostable Type II REases with different specificities have been discovered in species of the genus *Thermus *[15], including 17 prototypes: TatI [16], TauI [16], TaqI [17], TaqII [18], TfiI [19], TseI [20,21], TspDTI [22], TspGWI [23], Tsp4CI [24], TspEI [25], Tsp45I [26], TspRI [27], TstI [28], TsuI, TssI, Tth111I [29] and Tth111II [30]. Obviously, then, *Thermus *bacteria are a rich source of unique REases. A subset of these enzymes, the unique thermostable Type IIS REases from the *Thermus *sp. family, is worth mentioning. We have already published the criteria for defining the *Thermus *sp. family of REases [22]. Examples of the family include TspGWI [14,22,23,31], TspDTI [22], Tth111II [30], TthHB27I [15], TaqII [18] and TsoI [15], Arvydas Lubys, personal communication). The members of this group are bifunctional enzymes with REase and methyltransferase (MTase) activities within a single polypeptide (subtype IIC, which partially overlaps subtype IIS). All are stimulated by S-adenosyl methionine (AdoMet) or its structural analogues sinefungin (SIN) and S-adenosyl homocysteine (AdoHcy), but otherwise they behave like Type II enzymes [6]. These enzymes also exhibit some features of Types I and III, however.

The bifunctional TaqII enzyme is closely related to TspGWI, a prototype of the *Thermus *sp. enzyme family [31]. According to Barker et al. TaqII REase recognizes the 5'-GACCGA-3' and 5'-CACCCA-3' sites and cleaves 11/9 nucleotides downstream [18]. The enzyme generates a certain partial fragmentation pattern corresponding to cleavage at the target site [18]. Under the conditions tested previously, no complete TaqII digestion was obtained, either by native (isolated from *Thermus aquaticus *YT) or by recombinant enzyme [18]. This was probably due to preferential site cleavage in various DNA substrates. The restriction activity of TaqII is neither inhibited nor stimulated by ATP. Interestingly, in contrast to the TspGWI enzyme, TaqII REase displays marked differences in its response to AdoMet, which effectively stimulates its restriction endonuclease activity [22]. AdoMet is required for effective DNA methylation. Barker et al. noted minor endonuclease activity in TaqII preparation. However, the separation of this putative activity from TaqII in the course of purification was impossible, as native TaqII is present in *Thermus aquaticus *YT in minute amounts [18]. Purification is therefore complicated and results in low final preparation yields. In addition, non-specific nucleases, co-purifying with native TaqII, make precise digestions difficult. Further complications in the enzyme analysis stem from the fact that, like other enzymes from the *Thermus *sp. family, TaqII does not cleave DNA to completion to give a stable partial digestion pattern. After having noticed serious discrepancies between the experimentally obtained TaqII DNA digestion pattern (in the reaction buffer proposed by Barker et al.) and that of the recognition sequence published by Barker et al. [18], we decided to explore TaqII reaction features in greater detail. This paper has three aims: (*i*) to re-evaluate TaqII recognition and cleavage sites; (*ii*) to optimize both star-minimum and star-maximum reaction conditions for converting this prototype endonuclease, so far unused in recombinant DNA technology, into a useful molecular tool; (*iii*) to introduce the Fidelity Index for partially cleaving REases (FI-PC) - a useful variant of the Fidelity Index [32].

In this paper, we show that recombinant TaqII REase is unable to cleave after 5'-CACCCA-3' sites. Thus, the prototype recognition sequence should be redefined as cleaving after 5'-GACCGA-3' sites only. We determined the conditions both for minimizing and for stimulating star activity; we also introduced the Fidelity Index for Partial Cleavage (FI-PC).

## Results and discussion

### Devising artificial substrates differentiating TaqII recognition site variants

**Figure 1:**
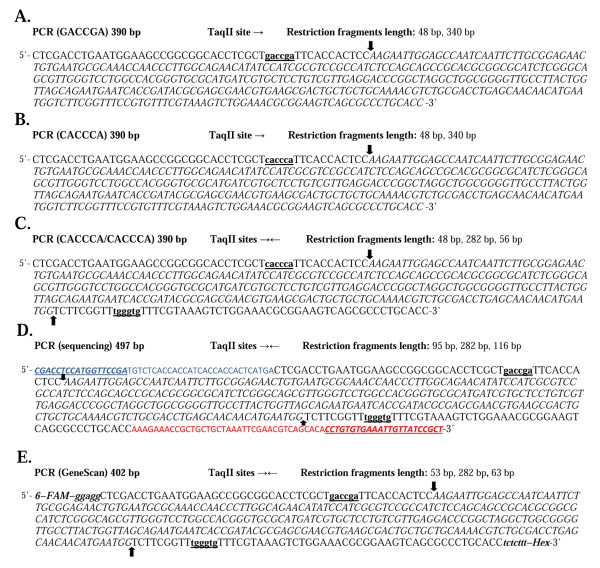
**PCR fragment DNA substrates**. Putative recognition sequences of TaqII are in bold and underlined. Arrows mark the cleavage. The restriction fragments lacking TaqII recognition sequence are in italics. (A) PCR DNA fragment with a single 5'-GACCGA-3' site. (B) PCR DNA fragment with a single 5'-CACCCA-3' site. (C) PCR DNA fragment with two 5'-CACCCA-3' sites. (D) PCR DNA fragment used for sequencing reactions. The distances from the 5'- and 3'-ends to the TaqII recognition sites were extended using an additional pair of primers. The introduced DNA fragments are shown in blue for the forward primer and in red for the reverse primer. (E) PCR DNA fragment used for GeneScan analysis. 6-FAM and Hex denotes the fluorescence dyes. The introduced DNA fragments are shown in bold and italics.

**Figure 2:**
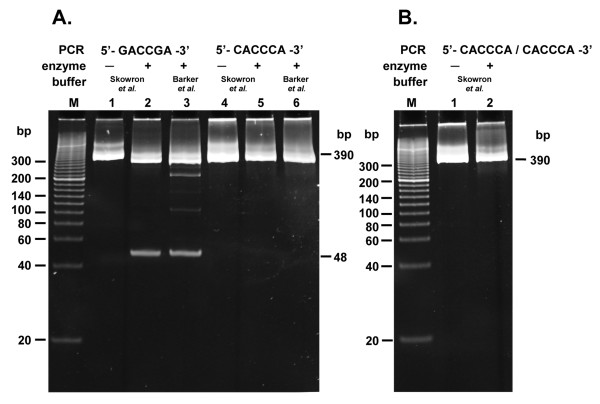
**TaqIl cleavage patterns of DNA substrates, containing 5'-CACCCA-3' and 5'-GACCGA-3' recognition sites**. (A) Digestion of the PCR DNA fragment with a single 5'-GACCGA-3' or 5'-CACCCA-3' site. Digestion of 0.3 μg (= 1.2-pmol recognition sites) PCR(GACCGA) substrate (lanes 1-3) and PCR(CACCCA) substrate (lanes 4-6) with 12 pmol TaqII, electrophoresed on a 15% polyacrylamide/TBE gel. Lane M, Sigma PCR 20-bp Low Ladder (selected bands marked); lane 1, undigested PCR(GACCGA) fragment; lane 2, digestion in the optimized buffer; lane 3, digestion in the buffer according to Barker *et al*. [18]; lane 4, undigested PCR(CACCCA) fragment; lane 5, digestion in the optimized buffer; lane 6, digestion in the buffer according to Barker *et al*. [18]. (B) TaqII digestion of the PCR DNA fragment with two convergent sites 5'-CACCCA-3'. Digestion of 0.3 μg (= 2.4-pmol recognition sites) PCR(CACCCA/CACCCA) substrate with 24 pmol TaqII. Lane M, Sigma PCR 20-bp Low Ladder (selected bands marked); lane 1, undigested PCR fragment; lane 2, digestion in the optimized buffer.

As both the native TaqII enzyme and that cloned into *Escherichia coli (E. coli) *(manuscript in preparation) exhibited the same cleavage pattern, the recombinant enzyme was selected for further work. Set up to explain the observed discrepancy between published [18] and observed TaqII specificity (not shown) using plasmid DNA substrates, the initial experiments were inconclusive, even though the cleavage patterns strongly suggested that the recognition sequence of this prototype differed from the sequence originally reported in 1984 [18]. Thus a set of five artificial DNA substrates from 390 to 402 bp in length was devised and obtained by PCR using a combination of mutagenic primers. The substrates contained selected canonical sites - variant 5'-CACCCA-3', variant 5'-GACCGA-3' or both - to eliminate/explore the potential stimulatory effect of two recognition sequences, present in the *cis *configuration, on the activity of TaqII REase (Figure 1). As shown in Figure 2A, the expected 48 bp and 340 bp restriction fragments from the digestion of two alternative DNA substrates, PCR(GACCGA) (Figure 1A) and PCR(CACCCA) (Figure 1B), containing a single (→) canonical site (either 5'-GACCGA-3' or 5'-CACCCA-3' [18], were obtained only for the first variant. Moreover, and typically of the TaqII mode of action (as it belongs to the *Thermus *sp. family, where all enzymes partially cleave DNA), TaqII digestion of PCR(GACCGA) was not complete. These results show clearly that 5'-GACCGA-3' site is efficiently cleaved, whereas the second 5'-CACCCA-3' canonical site, reported by Barker et al. [18], was not cleaved at all, as detected in a very sensitive assay using the Sybr Green staining procedure. It is interesting to note that in contrast to the homologous TspGWI enzyme, which recognizes a related recognition site and also cleaves at 11/9 nt away, TaqII REase is capable of single, isolated cognate site cleavage. The results were further confirmed by the incubation of PCR(CACCCA/CACCCA) 390 bp DNA substrate, containing two convergent 5'-CACCCA-3' sequences (Figure 1C). As expected, no digestion of the substrate was detected using Sybr Green staining (Figure 2B). The same results were invariably obtained under a variety of buffer conditions (not shown). The experiments with bacteriophage lambda DNA also addressed this issue, as multiple 5'-CACCCA-3' sequences are present there in all possible arrangements. Even so, no clone was found to have resulted from 5'-CACCCA-3' cleavage (Table 1).

**Table 1:** Specificity of TaqII REase.

TaqII recognition sequence variant	Theoretical number of TaqII recognition sequences in bacteriophage λ DNA	Number of cleaved TaqII recognition sequences (insert clones ends analysed)
5'-**GACCGA**-3'	10	39
5'-**CACCCA**-3'	18	--
Undetermined degenerate TaqII* star recognition sequences	?	59*

Determination of the TaqII recognition sequence and cleavage positions by shotgun cloning and sequencing of the resulting clones.

Relaxed recognition sites were determined by shotgun cloning and sequencing of the TaqII restriction fragments obtained. After digestion, DNA was blunted with T4 DNA polymerase and cloned into the *Sma*I site of the pUC19 vector. TaqII 5'-GACCGA-3' and TaqII 5'-CACCCA-3', canonical recognition sequences of the enzyme as described by Barker *et al*. [18]; TaqII*, variants of relaxed recognition sequences.

### Optimization of reaction conditions for TaqII REase activity

The PCR substrates made for the precise quantitative determination of the optimal reaction conditions (as defined by the minimum amount of the enzyme needed to obtain a stable DNA digestion pattern, regardless of any accompanying star activity). Since TaqII exhibits pronounced star activity, two variants of the reaction conditions were determined, resulting in (1) minimum star activity, while maintaining reasonable cleavage activity, and (2) maximum stimulation of star activity. The Fidelity Index could be calculated from a comparison of the TaqII digestion patterns obtained in the established reaction buffers (1) and (2), enabling the rational use of TaqII in recombinant DNA technology.

**Figure 3:**
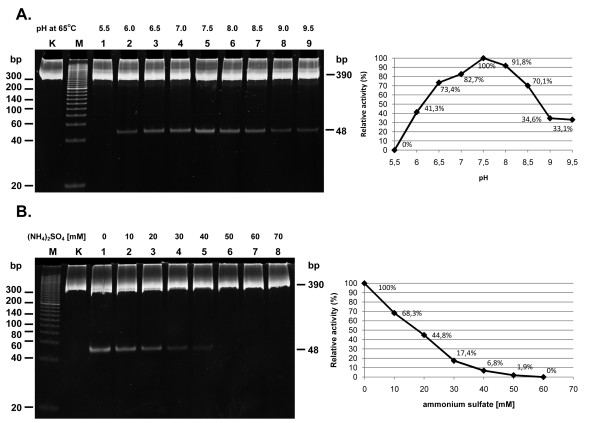
**Optimization of TaqII REase reaction conditions**. (A) The pH activity range of TaqII REase. 0.3 μg (= 1.2-pmol recognition sites) PCR(GACCGA) substrate was digested with 0.75 pmol TaqII in the pH range from 5.5 to 9.5 for 30 min. at 65°C. Lane M, Sigma PCR 20-bp Low Ladder (selected bands marked); lane K, undigested PCR fragment; lanes 1-9, digested PCR fragment: lane 1, at pH 5.5; lane 2, 6.0; lane 3, 6.5; lane 4, 7.0; lane 5, 7.5; lane 6, 8.0; lane 7, pH 8.5; lane 8, 9.0; lane 9, 9.5. The intensity of the 48 bp band on the series of gels was measured using UnScan-It software and the percentage of relative enzymatic activity calculated. (B) The influence of ionic strength on TaqII REase activity. 0.3 μg (= 1.2-pmol GACCGA recognition sites) PCR(GACCGA) substrate was digested with 2.4 pmol TaqII in the ammonium sulphate concentration range from 0 to 70 mM for 30 min. at 65°C. Lane M, Sigma PCR 20-bp Low Ladder (selected bands marked); lane K, undigested PCR fragment; lanes 1-8, digested PCR fragment: lane 1, without (NH_4_)_2_SO_4_; lane 2, with 10 mM (NH_4_)_2_SO_4_; lane 3, 20 mM; lane 4, 30 mM; lane 5, 40 mM; lane 6, 50 mM; lane 7, 60 mM; lane 8, 70 mM. The intensity of the 48 bp band was measured on a series of gels using UnScan-It software and the percentage of relative enzymatic activity calculated.

As a starting point, we used a previously published buffer composition [18]. Three buffering systems exhibiting the highest buffering capacity in the pH range tested were used for optimal pH determination: sodium acetate-acetic acid for pH from 5.0 to 5.5, HEPES-KOH for pH from 6.0 to 7.0, and Tris-HCl buffer for pH from 7.5-9.5. The pH of the reaction buffers was adjusted at 65°C after all the buffer components had been added. The cleavage reactions were performed under unsaturating conditions, where the enzyme was a limiting factor (0.625: 1 molar ratio of the enzyme to 5'-GACCGA-3' sites for 30 min). No restriction activity was detected in the 5.0-5.5 range; activity increased to values close to optimal (from 41.6 to 82%) in the 6.0-7.0 range, and reached a maximum in the 7.5-9.5 range at pH 7.5-8.0, while decreasing to 70% at pH 8.5, to 34% at pH 9.0 and to 33% at pH 9.5 (Figure 3A). At optimized pH (= 7.5), a buffer with variable concentrations of ammonium sulphate was used to determine optimal ionic strength. Ammonium sulphate was chosen, as we noticed the stabilizing effect of either NH_4_^+ ^or (SO_4_)^2- ^or both on TaqII enzyme, which translated into a longer lifetime of the enzyme in the reaction mixture and stronger suppression of star activity, as compared to NaCl or KCl (not shown). The maximum activity in the 0-70 mM concentration range tested was obtained between 0 and 10 mM (Figure 3B). As we did not observe any enzyme stabilization or activity increase with glycerol or non-ionic detergents, we did not evaluate these parameters further.

### Determination of the TaqII star inhibitory/stimulatory condition and the Fidelity Indices

**Figure 4:**
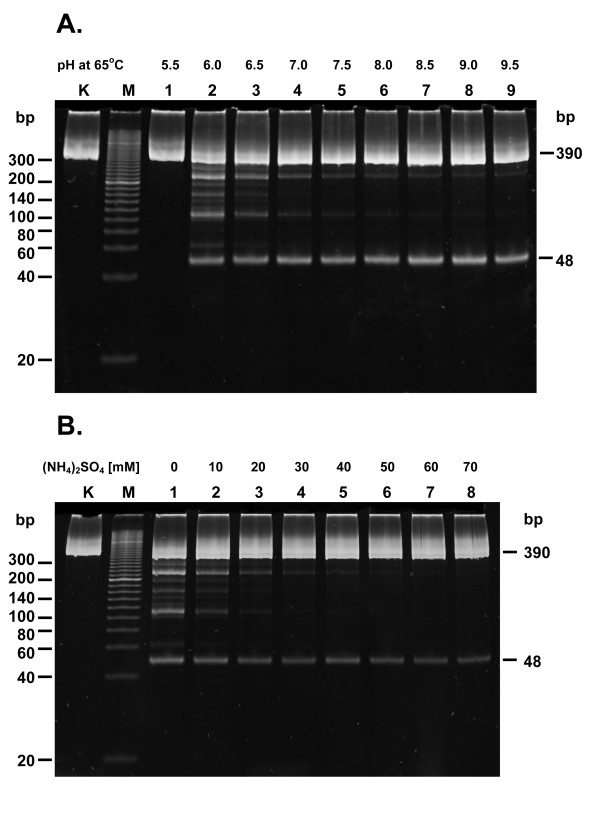
**Determination of reaction conditions minimizing and stimulating TaqII star activity**. (A) Influence of pH on TaqII star activity. 0.3 μg (= 1.2-pmol GACCGA recognition sites) PCR(GACCGA) substrate was digested with 12 pmol TaqII in the pH range from 5.5 to 9.5 for 16 h at 65°C in the reaction buffer: 40 mM of the appropriate buffering agent, 10 mM MgCl_2_, 1 mM DTT, BSA 100 μg/mL. Lane M, Sigma PCR 20-bp Low Ladder (selected bands marked); lane K, undigested PCR fragment; lanes 1-9, digested PCR fragment: lane 1, at pH 5.5, lane 2, 6.0; lane 3, 6.5; lane 4, 7.0; lane 5, 7.5; lane 6, 8.0; lane 7, 8.5; lane 8, 9.0; lane 9, 9.5. (B) Influence of ionic strength (ammonium sulphate) on TaqII star activity. 0.3 μg (= 1.2-pmol GACCGA recognition sites) PCR(GACCGA) substrate was digested with 12 pmol TaqII in the ammonium sulphate concentration range from 0 to 70 mM for 16 h at 65°C in the following reaction buffer: 40 mM Tris-HCl, pH 8.0 at 65°C, 10 mM MgCl_2_, 1 mM DTT, BSA 100 μg/mL. Lane M, Sigma PCR 20-bp Low Ladder (selected bands marked); lane K, undigested PCR fragment; lanes 1-9, digested PCR fragment: lane 1, without (NH_4_)_2_SO_4_; lane 2, with 10 mM (NH_4_)_2_SO_4_; lane 3, 20 mM; lane 4, 30 mM; lane 5, 40 mM; lane 6, 50 mM; lane 7, 60 mM; lane 8, 70 mM.

Since TaqII exhibits substantial star activity, it is crucial for potential applications of the enzyme in recombinant DNA technology to find the conditions minimizing this phenomenon. We had expected that the buffer conditions minimizing star activity might not necessarily match those of maximum activity, so we explored TaqII behaviour under enzyme oversaturating conditions to pinpoint subtle changes in star activity appearance; we also used a series of gel photographs, ranging from normal exposure to several stops of overexposure. The amount of enzyme activity used was effectively ca 400 times higher (assuming the confirmed overnight stability of TaqII, not shown) than that used for pH and ionic strength optima. In the pH range tested (5.0 to 9.5) star activity reached a maximum at pH = 6.0 and decreased sharply at pH = 7.5, which is also the value of general peak activity (Figure 4A). The results of the ionic strength effect are striking: there is nearly complete inhibition of star activity in 40-50 mM ammonium sulphate, which is far from the optimal activity value of 0-10 mM (Figure 4B). Thus we determined the minimum star activity conditions ("clean" DNA digestion for cloning technology) at pH 8.0 in 10 mM ammonium sulphate, while maximum star activity was obtained at pH 6.0 in 0 mM ammonium sulphate (no salt).

**Figure 5:**
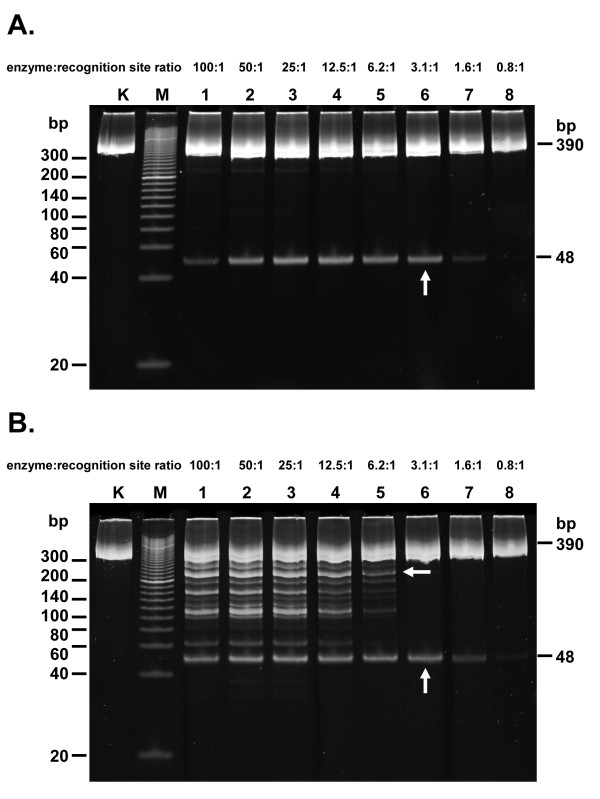
**Extending the Fidelity Index to REases partially cleaving DNA: determination of minimum and maximum values for TaqII**. (A) TaqII Fidelity Index under star inhibitory conditions. 0.3 μg (= 1.2-pmol GACCGA recognition sites) PCR(GACCGA) substrate was digested with decreasing amounts of TaqII for 1 h at 65°C in the following buffer: 40 mM Tris-HCl, pH 8.0, at 65°C, 10 mM (NH_4_)_2_SO_4_, 10 mM MgCl_2_, 1 mM DTT, BSA 100 μg/mL. The final glycerol concentration was 5%. Lane K, undigested PCR fragment; lane M, Sigma PCR 20-bp Low Ladder (selected bands marked); lanes 1-8, digested PCR fragment: lane 1: with 120 pmol TaqII; lane 2, 60 pmol; lane 3, 30 pmol; lane 4, 15 pmol; lane 5, 7.5 pmol; lane 6, 3.75 pmol; lane 7, with 1.87 pmol; lane 8, 0.94 pmol. (B) TaqII Fidelity Index under star stimulated conditions. 0.3 μg (= 1.2-pmol GACCGA recognition sites) PCR(GACCGA) substrate was digested with decreasing amounts of TaqII for 1 h at 65°C in the following buffer: 40 mM Tris-HCl, pH 6.0, at 65°C, 10 mM MgCl_2_, 1 mM DTT, BSA 100 μg/mL. The final glycerol concentration was 5%. Lane K, undigested PCR fragment; lane M, Sigma PCR 20-bp Low Ladder (selected bands marked); lanes 1-8, digested PCR fragment: lane 1: with 120 pmol TaqII; lane 2, 60 pmol; lane 3, 30 pmol; lane 4, 15 pmol; lane 5, 7.5 pmol; lane 6, 3.75 pmol; lane 7, 1.87 pmol; lane 8, 0.94 pmol.

These conditions were then used to carry out titration in order to determine and compare Fidelity Indices at both extremes. In order to apply the Fidelity Index [32] to TaqII, which gave a stable partial digestion pattern as the final reaction result, we needed to define a variant of the Fidelity Index for such REases. We established the Fidelity Index for Partial Cleavage (FI-PC) as the ratio of the maximum amount of enzyme showing no star activity to the minimum amount needed to obtain a stable partial digestion pattern. Figure 5A shows the titration under close to the originally reported conditions [18] of TaqII activity and under optimal (for non-star) buffer conditions: pH 8.0 and 10 mM ammonium sulphate. There were no digestions with a 0.78: 1 molar ratio of the enzyme to cognate site 5'-GACCGA-3', but a stable partial digestion pattern was obtained at 3.12: 1. TaqII is a bifunctional endonuclease-methyltransferase and it was observed to be a "slow" enzyme, just like other *Thermus *sp. family enzymes [14,22,23,31]. Nevertheless, when we extended the digestion time beyond the standard titration conditions presented in Figure 5A, substantial digestion was obtained at the molar ratio of 0.78: 1, indicating that TaqII is a multi-turnover enzyme (not shown). No star bands were detected until the ratio of 50: 1 was reached. The highest ratio tested (100: 1) evidently became inhibitory, though still leading to digestion and no star bands, possibly due to the formation of dominant non-specific REase-DNA complexes. Thus, FI-PC takes values of > 16-32 for the optimized conditions, which is close to or exceeds the FI value originally established as the Enzyme Class "good" [32]. This finding is important for practical applications of TaqII in recombinant DNA methodology, with minimal star activity interfering with the desired cleavage pattern. On the other hand, as can be seen in Figure 5B, very strong star activity was detected, dominating the expected non-star bands, under maximally relaxing conditions (pH 6.0, 0 mM ammonium sulphate). Under these conditions, FI-PC fell to 2 only. These conditions are actually also useful in DNA manipulation methods when applied to frequent cutter techniques, such as representative library preparations, RFLP, precise mapping, anonymous primer generation [33] or Thermal Cycle Labelling [34]. We are developing the "TaqII star" technology further (manuscript in preparation).

### Determination of TaqII recognition sequence and cleavage site

The most significant proof of the redefinition of the prototype TaqII specificity came from the digestion of custom devised substrates (Figure 1, Figure 2), containing both variants of the originally reported recognition sequence [18]. Those results have been confirmed with three additional independent methods: shotgun cloning of bacteriophage lambda DNA cut with TaqII and end-repaired, direct DNA sequencing of TaqII digestion products, and GeneScan analysis of the gel isolated TaqII restriction fragment. Since a large number of 5'-GACCGA-3' sequences (10 sites) and 5'-CACCCA-3' sequences (18 sites) are present in the bacteriophage lambda genome, this approach eliminates any bias towards potentially unfavourable nearest neighbourhood locations of the recognition sites and also the rather remote possibility of double divergent or convergent 5'-CACCCA-3' sequence arrangements being needed for cleavage, since all possible combinations of both sequence arrangements are present in lambda DNA. Even so, no cleavage is observed after 5'-CACCCA-3' sites.

For the shotgun cloning approach, the original Barker et al. [18] reaction buffer was used for a direct comparison of the results. Even though there is an excess of 5'-CACCCA-3' sequences over 5'-GACCGA-3' sequences in bacteriophage lambda DNA, 39 of the 98 insert ends in the clones analysed were DNA fragments resulting from 5'-GACCGA-3' site cleavage and none from 5'-CACCCA-3' sequence cleavage. Interestingly, in addition to the expected insert ends obtained, 59 ends are apparently due to star activity at 5'-GACCGA-3' sequences (Table 1), where no simple relaxation pattern is observed, as in the case of our previous report concerning related TspGWI [14]. This indicates that star activity, traditionally treated as an unwanted artifact, is in fact, for certain enzymes, a naturally inherent enzymatic activity (further work in progress). Even though the star minimizing buffer composition is much closer to physiological conditions *in vivo *than the star promoting condition, a strikingly high content of star cleaved insert ends is observed in the clones.

**Figure 6:**
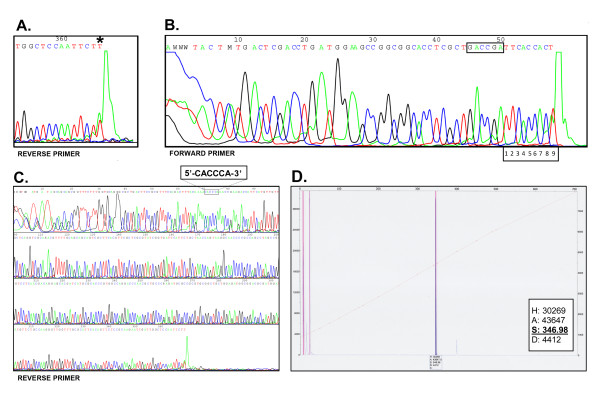
**Determination of TaqII REase recognition sequence and cleavage site**. Cleavage reactions of PCR(sequencing) and PCR(GeneScan) DNA substrates were performed as described in Materials and Methods. (A) This panel is the enlargement of the sequence end shown in Figure 6C. DNA sequencing of a 401 bp TaqII cleavage reaction product using reverse primer, annealing to the top strand, as arbitrarily set for the strand containing 5'-GACCGA-3' cleavable TaqII site (Figure 1D). The appropriate DNA fragment was isolated from an agarose gel following gel electrophoresis. The asterisk denotes the cleavage point at position 98 of the substrate DNA fragment (Figure 1D). (B) DNA sequencing of a 95 bp TaqII cleavage reaction product using forward primer, annealing to the bottom strand, as arbitrarily set for the strand containing 5'-GACCGA-3' cleavable TaqII site (Figure 1D). Thus the polymerization reaction goes through 5'-GACCGA-3' and stops at the end of the bottom strand template due to the cleavage distance of 9 nt. The appropriate DNA fragment was isolated from an agarose gel following gel electrophoresis. (C) DNA sequencing of non-purified TaqII cleavage reaction products using reverse primer, annealing to the top strand, as arbitrarily set for the strand containing 5'-GACCGA-3' cleavable TaqII site (Figure 1D). The sequencing reaction ends at the TaqII cleavage point at the 98 position of the substrate PCR(sequencing) DNA fragment (Figure 1D). (D) GeneScan gel analysis of TaqII cleavage. TaqII-cleaved 5'-labelled 402 bp PCR was gel purified and capillary electrophoreses in the presence of labelled DNA length markers. The TaqII cleavage product length reading of 347 nt (assuming standard +/- 1 nt error of the GeneScan) indicates 11/9 cleavage points after the 5'-GACCGA-3' site.

Direct confirmation of the previous findings came from the run-off sequencing approach. The PCR(sequencing) 497 bp DNA substrate (Figure 1D), containing convergent 5'-CACCCA-3' and 5'-GACCGA-3' sequences located on the same DNA fragment, was cleaved with TaqII REase and subjected to DNA sequencing with the dideoxy chain termination method utilizing fluorescent labelling in the α position. The PCR substrate generated was devised to serve as an internal control for TaqII cleavage and DNA sequencing - both cognate site variants were present in the same DNA molecule. The investigated sequences are clearly seen because of the distance of 11/9 nt between the cleavage site and the DNA recognition sequence (Figure 6AB). Unfortunately, owing to the known features of thermostable DNA polymerase already included in the pre-mixed sequencing kit routinely used by the commercial service that we employed, resulting in polymerization difficulties at the very end of a linear DNA template that are additionally complicated by the addition of adenines at the 3' end of the synthesized DNA, the precise cleavage point could not be reliably confirmed by the direct sequencing approach. Nevertheless, sequencing enabled the "clean" sequence to be read through the recognition sequence variant 5'-GACCGA-3', followed by DNA cleavage in the region of 11/9 nt downstream. On the other hand, the sequencing reaction goes through the putative cleavage region located 11/9 downstream from the recognition sequence variant 5'-CACCCA-3' without any decrease in signal strength (Figure 6B). These results clearly confirmed that TaqII neither recognizes nor cleaves the 5'-CACCCA-3' DNA sequence variant.

In order to establish the recognition site with an independent technique and to obtain final confirmation of the recognition of the variant 5'-GACCGA-3' as well as cleavage at 11 nt downstream in the top strand we used commercial GeneScan technology. Based on the capillary electrophoresis of a set of labelled standards of fixed length with 1-bp resolution, this method provides a simple and precise means of comparing the length of the TaqII cleavage products when electrophoresed together. This approach eliminates the above difficulties associated with the sequencing approach, where DNA polymerase does not yield a clean end sequence. For this purpose a TaqII substrate was prepared: this is a PCR fragment of 402 bp, containing both 5'-GACCGA-3' and 5'-CACCCA-3' sites, located at opposite ends of the DNA molecule (Figure 1E). The cleavage of the DNA substrate should result in the 53 bp, 282 bp and 63 bp restriction fragments (Figure 1E) if completely digested at both sequences. However, no complete TaqII digestion of the 402 bp PCR was obtained under the variety of conditions tested. A larger gel-isolated partial digestion fragment, the length of which is in the optimal range for GeneScan reading, was analysed. The value of 347 nt (+/- 1 nt as error range reported by the GeneScan service provider) thus obtained is in perfect agreement with 11/9 cleavage after the 5'-GACCGA-3' site variant.

The four approaches presented in this paper clearly show that TaqII cleaves only after 5'-GACCGA-3' sequences. This fundamental difference, as compared to the originally published prototype [18], may be due to a number of factors: (*i*) in 1984 DNA analysis technology was much less refined than nowadays, so the 5'-CACCCA-3' variant would have been an artifact of the technique; (*ii*) since TaqII's star activity is strong, some of the star sites could have been mistakenly assigned as 5'-CACCCA-3'; (*iii*) native TaqII is present in minute amounts in the *Thermus aquaticus *YT strain, along with substantial non-specific nuclease activity; (*iv*) co-purifying the third REase activity (5'-CACCCA-3'), in spite of the application of several chromatographic steps. Thus, taken together, crude and low concentration preparation could have led to false cognate site determination. Redefining the prototype specificity along with the quantitative Fidelity Index data and establishing compromise digestion conditions, where some of the enzyme's activity is sacrificed in exchange for "clean" cleavage with minimum star activity, provides a new, useful molecular cloning tool - a robust thermostable REase with unique specificity.

## Conclusions

*i*. The recognition DNA sequence of TaqII recombinant restriction endonuclease has been redefined as 5'-GACCGA-3' only.

*ii*. The cleavage site distance has been confirmed.

*iii*. The pH and salts (ammonium sulphate) optima were determined.

*iv*. The reaction conditions for inhibiting and stimulating the strong star activity of TaqII were established.

*v*. The redefinition of the TaqII recognition sequence together with the reduction/elimination of its star activity adds this enzyme to the palette of useful DNA cleavage specificities.

*vi*. The previously defined Fidelity Index [32] has been extended to REases, thus yielding a stable partial digestion pattern.

## Methods

### Bacterial strains, media and reagents

The *taqIIRM *gene was cloned in several configurations and used to produce the TaqII recombinant protein (manuscript in preparation). *Escherichia coli *BL21DE3 {F ^- ^*ompT hsdS_B _*(*r_B_^- ^m_B_^-^*) *gal dcm *(DE3)} (Novagen) was used for *taqIIRM *gene expression. *Escherichia coli *DH11S {*mcrA *Δ[*mrrhsdRMS*(rK^-^, mK^+^)-*mcrBC*] Δ(*lac-proAB*) Δ(*recA1398*) *deoR*, *rpsL*, *srl-thi*, *supE*/*F*' *proAB*+ *lacI*Q*Z*Δ*M15*} (Life Technologies, Gaithersburg, MD, USA) was used for the transformation of ligation mixtures and DNA propagation.

The Plasmid Mini, Gel-Out and Clean-Up DNA purification kits were from A&A Biotechnology (Gdańsk, Poland); SmaI restriction endonuclease and T4 DNA polymerase from Eurx Molecular Biology Products (Gdańsk, Poland); the PCR 20-bp Low Ladder from Sigma-Aldrich Poland; Taq DNA Polymerase, λ DNA and plasmids pBR322 and pUC19 from Vivantis Technologies (Shah Alam, Malaysia). The DNA sequencing, GeneScan and PCR primer synthesis were performed at Genomed (Warsaw, Poland). All other reagents were purchased from Sigma-Aldrich (St. Louis, MO, USA).

### TaqII activity unit definition

Since TaqII does not yield complete digestion, regardless of the amount of the enzyme used, one unit of TaqII enzymatic activity is defined as the minimum amount of the enzyme needed to yield a stable digestion pattern during 1 h at optimum temperature in optimum buffer, using 1 μg of pUC19 plasmid DNA.

### PCR fragment DNA cleavage assay

The TaqII recognition sites were created using a pair of primers introducing specific point mutations (the changed nucleotides are written in small letters) within the PCR fragment amplified from pBR322 plasmid DNA. Two alternative PCR fragments (390 bp): PCR(GACCGA) and PCR(CACCCA) containing single (→) originally published canonical sites for TaqII [18], either 5'-GACCGA-3' or 5'-CACCCA-3', were amplified using *Taq *DNA polymerase and a pair of primers: RTaqII 5'-GGTGCAGGGCGCTGACTTCC-3' and 1FTaqII 5'-TCGACCTGAATGGAAGCCGGCGGCACCTCGCT**gACcGA**TTCACCACT-3' or 2FTaqII 5'-TCGACCTGAATGGAAGCCGGCGGCACCTCGCT**cACccA**TTCACCACT-3', respectively. The cleavage pattern of the DNA substrates used, expected from published data [18], should result in the 48 bp and 340 bp restriction fragments (Figure 1AB). For the PCR(CACCCA/CACCCA) substrate, an additional 5'-CACCCA-3' site was introduced to the PCR(CACCCA) to generate a substrate containing two TaqII divergent recognition sites using reverse primer 2RTaqII 5'-GGTGCAGGGCGCTGACTTCCGCGTTTCCAGACTTTACGAAA**CAccCA**AACCGAAGA-3' (Figure 1C). TaqII cleavage of PCR fragments was carried out in the appropriate reaction buffer at 65°C. The TaqII cleavage reaction of the PCR DNA fragment was performed in the following buffers: 10 mM Tris-HCl, pH 7.5, at 65°C, 10 mM MgCl_2_, 1 mM DTT, BSA 100 μg/mL and in the reaction buffer proposed by Barker *et al*.: 6 mM Tris-HCl, pH 7.5, 6 mM MgCl_2_, 6 mM β-mercaptoethanol and 100 μg/ml gelatin [18]. The protein to DNA recognition site molar ratio differs depending on the experiment. The reaction volume was 50 μL. After 16 h, the reactions were quenched with phenol/chloroform, and DNA was ethanol-precipitated from the aqueous phase. The DNA precipitate was collected by centrifugation and dissolved in 10 mM Tris-HCl, pH 8.0, at 25°C. The products were analysed by electrophoresis in either 2% agarose or 15% polyacrylamide gels.

### λ DNA cleavage assay

Cleavage was carried out in the reaction buffer proposed by Barker *et al*. [18]. The reaction volume of 50 μL contained 0.3 pmol recognition sites 5'-GACCGA-3' and 3 pmol recombinant TaqII protein. The molar ratio of protein to DNA 5'-GACCGA-3' recognition sites was approximately 10:1, and that of protein to DNA 5'-GACCGA-3' and 5'-CACCCA-3' recognition sites was approximately 0.36:1. After 16 h, the digestion was quenched with phenol/chloroform, and DNA was ethanol-precipitated from the aqueous phase. The DNA precipitate was collected by centrifugation and dissolved in 10 mM Tris-HCl, pH 8.0, at 25°C. To determine the recognition sequence and cleavage sites, the DNA samples were treated with T4 DNA polymerase in the presence of dNTP and subjected to shotgun cloning. All concentrations of TaqII given here refer to the monomeric form of protein Mr 118, 500.

### Optimization of TaqII REase reaction conditions

The investigations into the influence of pH on recombinant TaqII/TaqII* REase activity were carried out for the range from 5.0 to 9.5. The pH of all the reaction buffers was determined at the reaction temperature (65°C). The following 40 mM biological buffers were used: sodium acetate buffer for pH from 5.0 to 5.5, HEPES buffer for pH from 6.0 to 7.0, and Tris-HCl buffer for pH from 7.5-9.5.

The TaqII cleavage reactions of the PCR (GACCGA) DNA substrate were performed at 65°C in the reaction buffers: 40 mM of the appropriate buffering agent, 10 mM MgCl_2_, 1 mM DTT, BSA 100 μg/mL. All the reaction mixtures contained 1.2 pmol recognition site and 0.75 pmol recombinant TaqII protein. The molar ratio of protein to DNA recognition sites was approximately 0.625:1. The reaction volume was 50 μL. After 30 min, the reactions were quenched with phenol/chloroform, and DNA was ethanol-precipitated from the aqueous phase. The DNA precipitate was collected by centrifugation and dissolved in 10 mM Tris-HCl, pH 8.0, at 25°C. The products were analysed by electrophoresis in either 2% agarose or 15% polyacrylamide gels.

The intensity of the band corresponding to the 48 bp TaqII restriction fragment was measured using UnScan-It software and the relative enzymatic activity was calculated as a percentage. The optimal reaction buffer was chosen and the influence of the increasing ionic strength on the reduction of TaqII* star activity was determined. Ammonium sulphate in the concentration range from 0 to 70 mM was added to the reaction buffer in order to obtain maximum TaqII activity with simultaneous elimination of TaqII* activity.

### Determination of TaqII cleavage site by DNA sequencing

For the PCR(sequencing) substrate, an additional 5'-CACCCA-3' site was introduced to the PCR(GACCGA) DNA fragment using the analogous reverse primer 2RTaqII 5'-GGTGCAGGGCGCTGACTTCCGCGTTTCCAGACTTTACGAAA**CAccCA**AACCGAAGA-3' (Figure 1D). The distances from the 5'- and 3'-ends to the TaqII recognition sites were extended using a pair of primers, complementary to the PCR (GACCGA/CACCCA) substrate, in the second round of the PCR reaction. The DNA sequences of the primers used were: F63-390 5'-CGACCTCCATGGTTCCGATGTCTCACCACCATCACCACCACTCATGACTCGACCTGAATGGAA-3' and R75-390 5'-AGCGGATAACAATTTCACACAGGTGTGCTGACGTTCGAATTTAGCAGCAGCGGTTTCTTTGGTGCAGGGCGCTGA-3'. The resulting PCR fragment was 497 bp in length (Figure 1D).

The TaqII cleavage of the PCR (sequencing) DNA substrate obtained was carried out in the appropriate reaction buffer at 65°C. The TaqII cleavage reaction was performed in the reaction buffer proposed by Barker *et al*.: 6 mM Tris-HCl, pH 7.5, 6 mM MgCl_2_, 6 mM β-mercaptoethanol and 100 μg/ml gelatin [18]. The reaction mixture contained 56 pmol GACCGA recognition sites and 112 pmol recombinant TaqII protein. The protein to DNA recognition site molar ratio was approximately 2:1. The reaction volume was 500 μL. After 3 h, the reactions were quenched with phenol/chloroform, and DNA was ethanol-precipitated from the aqueous phase. The DNA precipitate was collected by centrifugation and dissolved in 10 mM Tris-HCl, pH 8.0, at 25°C. The products were analysed by electrophoresis in either 2% agarose or 15% polyacrylamide gels and subjected to DNA sequencing on a 3730xl DNA Analyser using the oligonucleotides F18seq-390: 5'-CGACCTCCATGGTTCCGA- 3' and R22seq-390: 5'-AGCGGATAACAATTTCACACAGG-3' and BigDye^® ^Terminator v3.1 Applied Biosystems (Life Technologies).

### Determination of TaqII recognition site by shotgun cloning

The TaqII recognition site and cleavage positions were established by shotgun cloning and sequencing of the digestion products of bacteriophage λ DNA. The TaqII-generated restriction fragment ends were blunted with T4 DNA polymerase in the presence of dNTPs, cloned into the *Sma*I site of pUC19 vector, transformed into *E. coli *DH11S, and plated onto X-gal/IPTG plates [35]. Plasmid DNA from 50 different bacterial clones (from white colonies) was isolated, and the fragment/vector junctions were sequenced. The sequence data obtained were then analysed using ABI Chromas 1.45 software (Perkin Elmer Applied Biosystems, Monza, Italy) and DNASIS 2.5 software (Hitachi Software, San Bruno, CA, USA).

### GeneScan analysis

For the PCR(GeneScan) substrate, an additional 5'-CACCCA-3' site was introduced to the PCR(GACCGA) DNA fragment (see Materials and Methods, Determination of TaqII cleavage site by DNA sequencing). In the second round of the PCR reaction, the fluorescence dyes 6-FAM and Hex were added to the 5'- and 3'-ends of DNA fragment using a pair of primers, complementary to the PCR (GACCGA/CACCCA) substrate. The DNA sequences of the primers used were F6FAM-390: 5'-ggaggCTCGACCTGAATGGAA-3' and RHex-390 5'-aaagagaGGTGCAGGGCG-3'. The PCR (GeneScan) fragment obtained was 402 bp in length (Figure 1E).

The cleavage reaction mixture (500 μl) containing 6 mM Tris-HCl, pH 7.5 at 65°C, 6 mM MgCl_2_, 6 mM β-mercaptoethanol and 100 μg/ml gelatin 38 pM PCR (GeneScan) DNA substrate and 380 pM of TaqII protein were incubated at 65°C for 16 hours. The products were analysed by electrophoresis in either 2% agarose or 15% polyacrylamide gels. The 345 bp restriction fragment was isolated from agarose and subjected to GeneScan analysis using an ABI 3730xl DNA Analyzer and internal size standard: GeneScan ROX-500 (PE Applied Biosystems). The data obtained were analysed using PeakScanner v 1.0 software (Applied Biosystems).

### Polyacrylamide gel electrophoresis

Polyacrylamide gels (15%) were prepared in 1× Tris-Borate-EDTA (TBE) buffer [35]. The gels were visualized after staining with Sybr Green I using a 312-nm UV transilluminator and photographed with a SYBR Green gel stain photographic filter.

## Authors' contributions

AŻS conceived the project, coordinated its execution, participated in the design and interpretation of the experimental analyses, performed some of the experiments and drafted the manuscript. OŻ performed most of the experiments. KŚ purified TaqII REase. JJF performed some of the experiments and participated in the preparation of the figures. PMS was the author of the concept of the *Thermus *sp. enzyme family and FI-PC, participated in the design and interpretation of the experiments, coordinated the execution of the project and drafted the manuscript. All the authors read and approved the final manuscript.
